# Improving the physical healthcare of COVID-19 patients in inpatient psychiatric settings

**DOI:** 10.1192/bjo.2021.547

**Published:** 2021-06-18

**Authors:** Marissa Lewis, Karolos Dionelis, Miguel Vecida, Rebecca Phelps, Helen Hopwood, Lawrence Yong, Jason Ng, Sara Veeramah, Miranda Lloyd, Tom Clark

**Affiliations:** Oxleas NHS Foundation Trust

## Abstract

**Aims:**

COVID-19 can spread rapidly in psychiatric inpatient settings. Previous studies have found that patients have a higher risk of hospitalisation and death than adults in the community. The aim of this project was to improve the care of patients with COVID-19 in psychiatric inpatient settings.

**Method:**

A baseline audit was conducted of care COVID-19 patients received in wards that experienced outbreaks in January 2021 in a London Mental Health Trust. Clinical notes were reviewed for management plans, including clear documentation of risk of serious illness, frequency of vitals monitoring, and thresholds for escalation to medical teams.

A new protocol was subsequently developed and implemented at one inpatient unit: “COVID-19: Early Identification of Risk and Management”. This included an adjusted 4C mortality score to determine risk of deterioration, and schedules for observation monitoring based on this outcome. Each schedule specified separate frequencies of monitoring of critical observations (oxygen saturations, respiratory rate) and routine observations, thus minimising unnecessary staff exposure. It prompted venous thromboembolism (VTE) assessment and documentation of escalation criteria.

**Result:**

44 patients were identified across three working age (WAA, n = 29) and two older age (OA, n = 15) adult wards. 7.5% of WAA and 33.3% of OA patients were hospitalised. 20% of OA patients died following a positive test. 58% of patients had a documented management plan for COVID-19, but only 56% mentioned observation frequency, 19% escalation criteria, and 9% risk of serious disease. No patient received a repeat VTE assessment following diagnosis. The audit identified inconsistent approaches to COVID-19 management between wards, and found no relationship between risk of deterioration and frequency of observation monitoring. Following implementation of this protocol, 100% (n = 4) of patients had a robust plan for COVID-19 management, and 100% received a VTE assessment.

**Conclusion:**

The audit supported previous findings that psychiatric inpatients are at risk of serious COVID-19 infection. This highlights an urgent clinical and ethical need to optimise COVID-19 care in psychiatric inpatient settings. The results of this audit suggest that risk factors for severe infection and elements of routine care are not widely understood or implemented by clinical staff. Introducing evidence-based protocols to support clinicians in managing the physical healthcare of these patients may be one way of promoting best practice. The improvement in care observed in the pilot study has resulted in this protocol being rolled out across the Trust in an ongoing quality improvement project.

